# Benchmarking insecticide resistance intensity bioassays for *Anopheles* malaria vector species against resistance phenotypes of known epidemiological significance

**DOI:** 10.1186/s13071-017-2134-4

**Published:** 2017-04-20

**Authors:** Nelius Venter, Shȕné V. Oliver, Mbanga Muleba, Craig Davies, Richard H. Hunt, Lizette L. Koekemoer, Maureen Coetzee, Basil D. Brooke

**Affiliations:** 1 Centre for Emerging, Zoonotic & Parasitic Diseases, National Institute for Communicable Diseases, Johannesburg, South Africa; 2 Wits Research Institute for Malaria, School of Pathology, Faculty of Health Sciences, University of the Witwatersrand, Johannesburg, South Africa; 3 Tropical Diseases Research Centre, Ndola, Zambia

**Keywords:** Malaria vectors, Insecticide resistance bioassays, Resistance intensity

## Abstract

**Background:**

Insecticide use via indoor residual spraying (IRS) or treated nets is the primary method for controlling malaria vector populations. The incidence of insecticide resistance in vector populations is burgeoning globally making resistance management key to the design of effective malaria control and elimination strategies. Vector populations can be assessed for insecticide resistance using a binary (susceptible or resistant) classification based on the use of the standard WHO insecticide susceptibility assay for adult anopheline mosquitoes. However, the recent scaling up of vector control activities has necessitated a revision of the WHO bioassay protocol to include the production of information that not only diagnoses resistance but also gives information on the intensity of expression of resistance phenotypes detected. This revised protocol is expected to inform on the range of resistance phenotypes in a target vector population using discriminating/diagnostic insecticide concentrations (DC) as well as their potential operational significance using 5× DC and 10× DC assays. The aim of this project was to use the revised protocol to assess the intensity of pyrethroid resistance in a range of insecticide resistant *Anopheles* strains with known resistance mechanisms and for which there is evidence of operational significance in the field setting from which these colonies were derived.

**Methods:**

Diagnostic concentration (DC) bioassays followed by 5× DC and 10× DC assays using the pyrethroid insecticides permethrin and deltamethrin were conducted according to the standard WHO bioassay method against pyrethroid resistant laboratory strains of *Anopheles funestus*, *An. arabiensis* and *An. gambiae*.

**Results:**

Low to moderate resistance intensities were recorded for the *An. arabiensis* and *An. gambiae* strains while moderate to high intensities were recorded for the *An. funestus* strains.

**Conclusions:**

It is evident that resistance intensity assays can add predictive value to the decision making process in vector control settings, although more so in an IRS setting and especially when bench-marked against resistance phenotypes of known operational significance.

## Background

The effective control of malaria relies primarily on the suppression of vector mosquito populations. Currently, insecticide use is the only proven method by which to reduce the rate of malaria transmission to < 5 locally acquired cases per 1000 population at risk, the WHO definition for control [1]. Enhancing this effect towards the successive phases of pre-elimination (<1 case/1000 population at risk), elimination (0 local cases/1000 population) and ultimately eradication requires an integrated vector management approach that is not solely based on the use of insecticides. Nevertheless, the use of insecticides, either through indoor spraying of residual insecticides (IRS) or the distribution of long-lasting insecticidal bed nets (LLINs) or both, is necessarily the core component of vector control for all phases of the global malaria eradication programme [2, 3].

The burgeoning global incidence of insecticide resistance in target vector populations [4] has led to a situation in which insecticide based control has become synonymous with the necessity for resistance management [5]. This is especially pertinent in settings where it has been demonstrated that resistance has caused control failure [6–8]. The Global Plan for Insecticide Resistance Management (GPIRM) [9] and the Integrated Vector Management (IVM) strategy [10] provide frameworks for establishing effective vector control programmes at local and regional levels despite the occurrence of resistance. In both instances, the need for coordinated and intensive vector surveillance is highlighted. This is because the design of an effective vector control programme for any target region relies on information concerning the spatial and temporal distribution of vector species and their insecticide susceptibility profiles as well as an assessment of the drivers of residual transmission where pertinent.

In most instances, insecticide resistance phenotypes are constructed using an array of physiological, neuronal and structural mechanisms. Metabolic resistance mechanisms based on the detoxifying properties of monooxygenase, glutathione S-transferase and esterase enzyme systems are necessary for the metabolic turnover of insecticide intoxication while reduced sensitivities of neuronal target sites can significantly reduce insecticide susceptibilities in certain combinations [11, 12]. Examples of these in malaria vector mosquitoes are the array of *kdr* mutations at position 1014 of the voltage-gated sodium gene that reduce sensitivity to DDT and certain pyrethroid insecticides, the *rdl* mutation in the GABA receptor that confers resistance to dieldrin and fipronil, and the *Ace-1* mutation in the acetylcholinesterase-1 gene that confers resistance to organophosphates and carbamates. Structural mechanisms such as cuticle thickening associated with resistance phenotypes [13] can reduce sensitivity and enhance the expression of resistance by slowing the rate of insecticide penetration across the cuticle [14].

In the core definition of resistance, vectors can be differentiated as either resistant or susceptible in a relative manner in which a resistant insect can survive a dose of toxicant that would normally kill all susceptible members of the same population and species [15]. It is from this definition that diagnostic doses/concentrations of public health insecticides were designed to test for the occurrence of resistance in target populations. However, this definition does not link resistance to the potential for operational control failure because there is no correlation between the diagnostic concentrations used for resistance bioassays and the concentrations of insecticide used to treat walls and fabrics. In addition, the diagnostic bioassay does not take the intensity of resistance expression into account.

Diagnostic insecticide concentrations for *Anopheles* species are usually given as twice the concentration per insecticide that induces 99.9% mortality in insecticide susceptible laboratory populations following a fixed period of exposure [15]. Based on these concentrations and using the standard WHO insecticide susceptibility assay for adult anophelines, a vector population can be assessed for the occurrence of insecticide resistance using a binary (susceptible or resistant) classification. This method has formed the basis of successive WHO guidelines for assessing insecticide susceptibility in adult anophelines over the past four decades and has enabled an evidence-based approach to resistance management [15–18]. However, the scaling up of insecticide-based malaria vector control over the past 15 years and the introduction of malaria elimination campaigns in several endemic countries has necessitated a revision of the WHO bioassay protocol to include the production of higher resolution information that not only diagnoses resistance but also gives information on the intensity of expression of any resistance phenotypes detected [19]. The WHO susceptibility test for adult mosquitoes has thus recently been expanded to include the use of discriminating concentrations, followed by 5× and 10× concentrations in a step-wise manner to provide information on the range (if any) of resistance phenotypes present in a target vector population and their potential operational significance [20]. This assay is relatively similar to the CDC Resistance Intensity Rapid Diagnostic test (I-RDT) [21] (except that the end points of the two methods differ) and is designed to provide a simple, practical method of assessing resistance intensity, especially under field conditions.

It is now necessary to bench-mark data obtained using diagnostic and intensity concentrations against insecticide resistant *Anopheles* populations in which the mechanisms of resistance and their operational significance is known. The aim of this project was therefore to use the expanded WHO bioassay protocol [20] to assess the intensity of pyrethroid resistance in a range of insecticide resistant *Anopheles* strains with known resistance mechanisms and for which there is evidence of operational significance in the field setting from which these strains were derived.

## Methods

### *Anopheles* laboratory strains

Laboratory strains used for resistance intensity assessments by species, origin, resistance profile and associated resistance mechanisms are described in Table 1. All strains are maintained under standard insectary conditions of 80% (± 5%) relative humidity, 25 °C (± 2 °C) ambient temperature and a 12-h day/night cycle with 45 min dusk/dawn transitions. Note that all ZAMF and KZN samples used in the resistance intensity experiments were the F1 progeny of wild-caught females.

**Table 1:** *Anopheles* spp. laboratory strains by species, origin, date of establishment, insecticide resistance profile and known resistance

*Anopheles* spp.	Laboratory strain	Origin and date of culture establishment	Resistance profile	Known resistance mechanisms
*An. funestus*	FUMOZ BASE & FUMOZ-R^a^	Southern Mozambique (2000)	Pyrethroids; carbamates	Monooxygenase P450s; glutathione S-transferase (secondary); thickened cuticles (secondary) [13, 23–27]
	ZAMF^b^	Nchelenge, Zambia (2016)	Pyrethroids; carbamates	Monooxygenase; P450s [28]
*An. arabiensis*	SENN DDT	Sennar, Sudan (1980)	Pyrethroids; DDT; organophosphates	Monooxygenase; P450s; glutathione S-transferase; general esterases; L1014F *kdr* [46, 47]
	MBN DDT	Northern KwaZulu-Natal, South Africa (2002)	Pyrethroids; DDT	Monooxygenase; P450s [34, 47, 48]
	KZN^b^	Northern KwaZulu-Natal, South Africa (2016)	Pyrethroids; DDT	Monooxygenase; P450s [34, 47, 48]
*An. gambiae*	TONGS	Tongon mine, Cote d’Ivoire (2010)	Pyrethroids; DDT; organophosphates; carbamates	Unknown

^a^FUMOZ-R was intensively selected for pyrethroid resistance from FUMOZ BASE

^b^All ZAMF and KZN samples used in the resistance intensity experiments were the F1 progeny of wild-caught females

### WHO susceptibility tests with diagnostic concentrations (DC)

Diagnostic concentration (DC) bioassays for the pyrethroid insecticides permethrin and deltamethrin were conducted according to the standard WHO bioassay method [18]. Samples of 3–5 day-old adult female mosquitoes were drawn from each strain and were exposed to filter papers treated with the diagnostic concentrations of either permethrin or deltamethrin (Table 2). At least 100 females per strain per insecticide, divided into at least four replicates of 20–25 females per tube, were exposed unless otherwise stated. Controls consisted of simultaneous exposures of samples of the same strain to solvent treated papers. All exposures were for 1 h, and final mortality was scored after a 24 h holding period during which a 10% sucrose solution was made available to surviving mosquitoes. All DC treated papers were procured from the WHO supplier at Universiti Sains Malaysia.

**Table 2:** Pyrethroid insecticides used for WHO susceptibility tests

Insecticide	Diagnostic concentration (DC)	5× DC	10× DC
Permethrin	0.75%	3.75%	7.5%
Deltamethrin	0.05%	0.25%	0.5%

### WHO susceptibility tests with intensity concentrations (5× and 10× DC)

5× and 10× DC bioassays for permethrin and deltamethrin were also conducted according to the standard WHO bioassay method as described above. All exposures were also for 1 h, and final mortality was scored after a 24 h holding period during which a 10% sucrose solution was made available to surviving mosquitoes. The following interpretation parameters were used as a guide [20]:

- Mortality in the range 98–100% at the 5× dose indicates that it is not necessary to assay at the 10× dose. A low resistance intensity is indicated.
- Mortality of less than 98% at the 5× dose indicates a moderate resistance intensity. It is necessary to assay further at the 10× dose.
- Mortality in the range 98–100% at the 10× dose confirms a moderate resistance intensity.
- Mortality of less than 98% at the 10× dose indicates high resistance intensity.

Filter papers treated with the 5× and 10× concentrations (Table 2) were prepared at the Vector Control Reference Laboratory of the National Institute for Communicable Diseases in Johannesburg. Technical-grade permethrin and deltamethrin (Sigma-Aldrich, St Louis, USA) were procured and diluted to working solutions in acetone and olive oil (1:1) according to the standard operating procedure supplied by Universiti Sains Malaysia. Wattmans no. 1 filter papers (12 × 15 cm) were treated with insecticide to the required concentration by hand pipetting 2 ml of the required working solution onto each paper in a spiral rotation. Control papers were treated with a 2 ml acetone/olive oil (1:1) solution. All treated papers were air-dried for at least 24 h before use. No treated paper was used more than three times.

## Results

Control mortalities in all experiments were < 5%, and so no data were adjusted [20]. According to the standard WHO criteria, all strains tested showed resistance to deltamethrin and permethrin-based on the use of discriminating concentrations (DC) (Tables 3, 4). Subsequent use of the 5× and 10× DC concentrations showed that the *An. funestus* FUMOZ BASE strain showed moderate resistance intensity to deltamethrin and low-intensity resistance to permethrin; the *An. funestus* FUMOZ-R and ZAMF strains showed high-intensity resistance to deltamethrin and moderate resistance intensity to permethrin; the *An. arabiensis* SENN DDT and MBN DDT strains showed low-intensity resistance to deltamethrin and moderate resistance intensity to permethrin; the *An. gambiae* TONGS strain showed low-intensity resistance to deltamethrin and moderate resistance intensity to permethrin (Tables 3, 4).

**Table 3:** Overall percentage mortalities for deltamethrin by species and strain based on the use of the standard WHO bioassays at the discriminating (DC), 5× and 10× concentrations. The sample sizes (*n*) and associated numbers of replicates are given in parentheses. Resistance intensity for each strain is classified as low, moderate or high based on revised criteria

Species	Strain	Overall % mortality (*n*; no. replicates)			Resistance intensity
		DC	5× DC	10× DC	
*An. funestus*	FUMOZ BASE	8 (100; 4)	89.13 (92; 4)	100 (100; 4)	moderate
	FUMOZ-R	4 (100; 4)	50.81 (124; 5)	87.25 (102; 4)	high
	ZAMF	23.68 (228; 8)	35.9 (39; 2)	80 (25; 1)	high
*An. arabiensis*	SENN DDT	54 (100; 4)	100 (103; 4)	–	low
	MBN DDT	80 (100; 4)	100 (100; 4)	–	low
	KZN	88 (75; 3)	100 (76; 3)	–	low
*An. gambiae*	TONGS	91 (100; 4)	100 (109; 4)	–	low

**Table 4:** Overall percentage mortalities for permethrin by species and strain based on the use of the standard WHO bioassays at the discriminating (DC), 5× and 10× concentrations. The sample sizes (*n*) and associated numbers of replicates are given in parentheses. Resistance intensity for each strain is classified as low, moderate or high based on revised criteria

Species	Strain	Overall % mortality (*n*; no. replicates)			Resistance intensity
		DC	5× DC	10× DC	
*An. funestus*	FUMOZ BASE	14.71 (102; 4)	98.92 (93; 4)	100 (100; 4)	low
	FUMOZ-R	6 (100; 4)	60 (100; 4)	100 (100; 4)	moderate
	ZAMF^a^	–	76 (17; 1)	100 (17; 1)	moderate
*An. arabiensis*	SENN DDT	30 (100; 4)	89.04 (146; 6)	100 (104; 4)	moderate
	MBN DDT	67.33 (101; 4)	86 (100; 4)	100 (100; 4)	moderate
	KZN	72 (74; 3)	82.43 (74; 3)	100 (49; 2)	moderate
*An. gambiae*	TONGS	69.9 (103; 4)	87 (100; 4)	100 (100; 4)	moderate

^a^Owing to a lack of samples, ZAMF was not assayed for resistance using the discriminating concentration for permethrin

## Discussion

Although all strains tested were classified as pyrethroid resistant based on the use of discriminating concentrations, the use of follow-on intensity assays showed that the response to pyrethroid intoxication was measurably variable between strains and species, and between type I (permethrin) and type II pyrethroids (deltamethrin). Given that the intensity of resistance expression is dependent on the characteristics of the underlying mechanisms, it is interesting to note that monooxygenase P450s have been implicated in resistance in all strains tested (except for TONGS), suggesting marked variation in the transcribed quantities and detoxifying capabilities of these enzymes, and marked variation in the contributions from other mechanisms to production of the resistance phenotypes. This variation can be described within the context of differences between detoxifying enzyme networks termed the pyrethrome [22].

The only strains showing high resistance intensity were *An. funestus* FUMOZ-R and ZAMF. This result is especially pertinent for various reasons. Primarily, the pyrethroid-carbamate resistance in FUMOZ-R has been thoroughly characterised and has been shown to be primarily based on the detoxifying properties of at least two P450s [23–26]. Co-factors include enhanced GST-mediated protection against the damaging effects of oxidative stress [27] and thickened cuticles in resistant adult females [13]. Bioassay and synergist assay experiments suggest that the similar pyrethroid-carbamate resistance profile in Zambian *An. funestus* [28] is mediated by the same mechanisms. The pyrethroid resistant phenotype produced by these mechanisms in *An. funestus* has been shown to cause operational failure in an IRS setting. Pyrethroid resistance was causally implicated in the malaria epidemic experienced in South Africa during the period 1996–2000. Before 1996, South Africa’s IRS based vector control programme was dependent on DDT [7]. In 1995, a policy to move away from the use of DDT for IRS in favour of pyrethroids was adopted. A primary cause of the epidemic that followed was the range expansion of pyrethroid resistant *An. funestus* following the introduction of pyrethroids for IRS [6, 7]. The re-introduction of DDT for IRS in South Africa post-2000 and the subsequent substantial decline in malaria incidence strongly suggests that pyrethroid efficacy was severely undermined by the development of pyrethroid resistance in *An. funestus* and that DDT use, in conjunction with pyrethroids, was necessary to re-establish control [6]. Thus, the high resistance intensity in FUMOZ-R can be linked to an instance of operational failure and therefore provides an opportunity to benchmark the intensity bioassays regarding their operational significance. Based on this principle, it can be predicted that the equally high resistance intensity recorded in Zambian *An. funestus* from Nchelenge using time exposures [28] and ZAMF in this study (both of which were based on the use of F1 progeny from wild-caught females and can, therefore, be suitably representative of the wild population), is likely to lead to operational failure of a pyrethroid-based IRS programme in this region. This prediction is borne out by studies published recently describing the malaria epidemiology and vector bionomics in Nchelenge [29, 30]. Zambia has revised its pyrethroid-based IRS policy to include the use of organophosphate insecticides because of burgeoning pyrethroid resistance in target vector populations, especially *An. funestus* [31].

The use of 5× and 10× diagnostic concentrations as a practical method for assessing resistance intensity is supported by other data sets. For example, high-intensity pyrethroid resistance in southern African *An. funestus* has been recorded using various methods. The intensity in FUMOZ BASE, FUMOZ-R and wild-caught Zambian *An. funestus* has been assessed using single diagnostic concentrations with prolonged exposure over time [28, 32]. These assays showed the significant survival of adult female mosquitoes against diagnostic concentrations even after 8 h continuous exposure. Furthermore, by using a range of permethrin concentrations and the CDC bottle bioassay method it has previously been established that the expression of permethrin resistance in FUMOZ-R is approximately 70-fold higher than the level of permethrin tolerance in the insecticide susceptible *An. funestus* FANG strain that originates from southern Angola [33]. All three methods for assessing resistance intensity (5× and 10× DC, concentration range and single dose time-response) have indicated a formidable pyrethroid resistance phenotype in southern African *An. funestus* that has been shown to have serious operational implications for malaria vector control in this region.

The potential operational significance of the low and moderate intensity phenotypes recorded for the *An. arabiensis* and *An. gambiae* strains used in these tests is more difficult to assess. The MBN DDT strain has been in culture since 2002 and is therefore not necessarily representative of the current perennial *An. arabiensis* population at Mamfene, northern KwaZulu-Natal, from which it originates. The wild-caught KZN strain, however, can be considered representative of the current population at Mamfene. The combined KZN and MBN DDT results are congruent with the low-level pyrethroid resistance that was recently recorded in this population [34] and suggest that this phenotype is unlikely to be causing an operational problem at present. This is because the incidence of locally acquired malaria in the Mamfene region is currently very low despite the occurrence of comparatively high numbers of potential vectors [35, 36]. However, it should be noted that the low malaria incidence in the Mamfene region may be due to a highly-diminished *Plasmodium* parasite population which may, in turn, be courtesy of the parasite clearance programme which has been initiated there [37]. Furthermore, live samples of *An. arabiensis* were collected in Mamfene in 2014 and 2015 using exit window traps attached to houses that had previously been sprayed with the pyrethroid alpha-cypermethrin [36], and their survival may be linked to resistance.

Pyrethroid resistance in *An. arabiensis* is widespread in the Gezira state of Sudan which is adjacent to the Sennar state from which SENN DDT was derived [38]. The use of pyrethroids for IRS in Gezira was discontinued in favour of the carbamate bendiocarb following assessments of pyrethroid resistance in *An. arabiensis* there in 2007 [39]. Although pyrethroid resistance has also been recorded in *An. arabiensis* in Khartoum state [40], there is currently no published information on the operational significance of pyrethroid resistance in *An. arabiensis* in Sudan although a large-scale study is currently underway to determine this [41]. Given that the DC percentage mortality for SENN DDT was far lower than MBN DDT for both deltamethrin and permethrin (100 and 89% mortality was achieved at 5× DC, respectively), it is suggested that operational failure should not be an issue in Sennar either. This remains to be confirmed by the field studies [41].

Pyrethroid resistance in *An. gambiae* is widespread in Cote d ‘Ivoire including the northern region [42] in which the Tongon mine is located and from which the TONGS strain was derived. The primary method of vector control in this region is the distribution of LLINs. Although the effect of low/moderate resistance intensity on LLIN efficacy is difficult to predict, it is notable that Cote d ‘Ivoire experienced a dramatic increase in malaria incidence during the period 2010–2013 which subsequently plateaued in 2014 and 2015 [43]. Insecticide resistance may partially account for this trend. This is somewhat reinforced by the results of a case study in northern Cameroon which showed that low/moderate intensity deltamethrin resistance, primarily in *An. arabiensis* can lead to a reduction in LLIN efficacy after more than ten washes [44]. Nevertheless, a meta-analysis by Strode et al. [45] concludes that insecticide treated nets (ITNs) can be an effective form of vector control despite insecticide resistance, as indicated by entomological outcomes. This is because ITNs are more effective in terms of reducing blood-feeding and killing mosquitoes than untreated nets even against a backdrop of insecticide resistance in target vector populations.

## Conclusions

It is evident that resistance intensity assays can add predictive value to the decision-making process in vector control settings, although more so in an IRS setting and especially when bench-marked against resistance phenotypes of known operational significance. Although it is easier to conduct intensity assays using a single concentration time-response method, it has been shown that this method is not suitable for highly resistant populations [19]. The use of discriminating concentrations followed by 5× and 10× concentrations as indicated in a stepwise manner provides a useful method of predicting the possible effect that a resistance phenotype may have concerning operational malaria vector control.

## Abbreviations

CDC I-RDT: Centers for Disease Control Resistance Intensity Rapid Diagnostic test
DC: Diagnostic concentration
DDT: Dichlorodiphenyltrichloroethane
GPIRM: Global Plan for Insecticide Resistance Management
GST: Glutathione S-transferase
IRS: Indoor residual spraying
ITNs: Insecticide treated nets
IVM: Integrated vector management
LLINs: Long-lasting insecticidal nets
WHO: World Health Organization
